# Analysis of the Serum Peptidomics Profile for Cats With Sarcomeric Gene Mutation and Hypertrophic Cardiomyopathy

**DOI:** 10.3389/fvets.2021.771408

**Published:** 2021-11-08

**Authors:** Pratch Sukumolanan, Narumon Phanakrop, Siriwan Thaisakun, Sittiruk Roytrakul, Soontaree Petchdee

**Affiliations:** ^1^Veterinary Clinical Studies Program, Faculty of Veterinary Medicine, Graduated School, Kasetsart University, Nakorn Pathom, Thailand; ^2^Functional Ingredients and Food Innovation Research Group, National Center for Genetic Engineering and Biotechnology, National Science and Technology Development Agency, Pathum Thani, Thailand; ^3^Proteomics Research Laboratory, National Center for Genetic Engineering and Biotechnology, National Science and Technology Development Agency, Pathum Thani, Thailand; ^4^Department of Large Animal and Wildlife Clinical Sciences, Faculty of Veterinary Medicine, Kasetsart University, Nakorn Pathom, Thailand

**Keywords:** feline, hypertrophic cardiomyopathy, myocardial disease, peptidomics analysis, echocardiography

## Abstract

**Background:** Hypertrophic cardiomyopathy (HCM) has a complex phenotype that is partly explained by genetic variants related to this disease. The serum peptidome profile is a promising approach to define clinically relevant biomarkers. This study aimed to classify peptide patterns in serum samples between cats with sarcomeric gene mutations and normal cats.

**Materials and Methods:** In the total serum samples from 31 cats, several essential proteins were identified by peptidomics analysis. The 5,946 peptides were differentially expressed in cats with sarcomeric gene mutations compared with cats without mutations.

**Results:** Our results demonstrated characteristic protein expression in control cats, Maine Coon cats, and Maine Coon cats with gene mutations. In cats with gene mutations, peptide expression profiling showed an association with three peptides, Cytochrome 3a132 (CYP3A132), forkhead box O1 (FOXO1), and ArfGAP, with GTPase domains, ankyrin repeats, and PH domain 2 (AGAP2).

**Discussion:** The serum peptidome of cats with mutations might provide supporting evidence for the dysregulation of metabolic and structural proteins. Genetic and peptidomics investigations may help elucidate the phenotypic variability of HCM and treatment targets to reduce morbidity and mortality of HCM in cats.

## Introduction

Myocardial disease is the most common genetic heart problem in cats, especially in Maine Coon, Ragdoll, and Persian cats. Hypertrophic cardiomyopathy (HCM) is one of the most common genetic heart problems in cats. HCM is a myocardial disease caused by dominant mutations in genes encoding cardiac sarcomere protein. Many gene polymorphisms, such as MYBPC3-A31P and A74T, have been detected in Maine Coon cats or cats crossbred with Maine Coon cats (1–4). HCM can be characterized by an increase in left ventricular myocardial mass. However, other causes of cardiac hypertrophy, such as hyperthyroidism, systemic hypertension, and aortic stenosis, are the primary differential diagnosis for this disease (5–7).

The complex and dynamic pathophysiological mechanisms surrounding cardiac hypertrophy have focused on many investigations seeking therapeutic strategies (8). Additional studies may involve understanding the risk factors for cardiovascular disease. Many studies have investigated the pathway that plays critical roles in mediating cardiac hypertrophy (9–12). The mechanisms for the effect of testosterone on muscle hypertrophy are not entirely understood. It is known that free testosterone concentrations increase in response to cardiac muscle hypertrophy (13). Previous studies have suggested that testosterone-induced cardiomyocyte hypertrophy is accompanied by increased glucose uptake and glycolysis. In addition, testosterone increased AMPK phosphorylation, which is the crucial pathway in the development of eccentric hypertrophy (14).

Peptide analysis is being increasingly used to identify the protein changes accompanying changes in clinical appearance. Two-dimensional gel electrophoresis coupled with mass spectrometry offers a key advantage of profiling proteins in biological samples. Alterations in the proteome profile determined using two-dimensional gel electrophoresis in conjunction with matrix-assisted laser desorption ionization-time of flight (MALDI-TOF) mass spectrometry may explain the mechanistic pathways and identify novel potential biomarkers for the diagnosis and prognosis of HCM in cats. The proteomic data may contribute to understanding the pathogenesis of myocardial remodeling, and the proteins found may be candidate biomarkers for the diagnosis and prognosis of HCM in cats.

The purpose of this study is to provide important information on clinical presentation, protein expression, and genes associated with feline HCM. The detection of HCM with a normal phenotype in the early stage may provide an advantage for the treatment strategies, prognosis, and prevention of HCM in the clinic.

## Materials and Methods

### Animals

**Figure 1:**
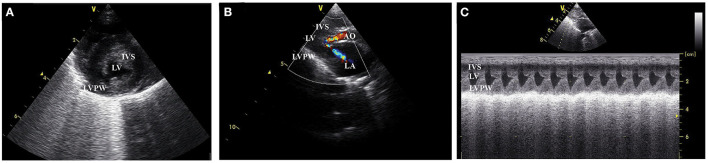
Left ventricular wall of HCM cat visualized by two dimensional echocardiography in the **(A)** short axis view, **(B)** color flow doppler in long axis view, and **(C)** the motion mode (M mode) in long axis view. IVS, interventricular septum; LVPW, left ventricular proximal wall; LV, left ventricle; LA, left atrium; AO, aorta.

This study design is a case-control study. The study protocol was approved by the Ethics Committee, Kasetsart University (ACKU-62-VET-059), and written informed consent from the owners. Thirty-one cats, 17 domestic shorthair cats, and 14 Maine Coon cats were enrolled in this study. The cat underwent a complete physical examination to evaluate the general condition. A complete blood count and serum biochemistry profile were performed for each cat. The cardiovascular system is evaluated by echocardiographic analysis. B-mode and M-mode echocardiography were used to define the left ventricular structure. The thickening of left ventricular wall at diastole phase which more than 6 mm indicated left ventricular hypertrophy (15). Echocardiography images have demonstrated left ventricular hypertrophy, the dynamic obstruction of the left ventricular outflow tract, which are common findings in HCM cats, as shown in Figure 1.

### DNA Sequencing

DNA amplification was performed to detect MYBPC3-A31P and A74T. The PCR product was extracted and purified with FavorPrep GEL/PCR Purification Kit, Taiwan. The purified PCR product was stored at −20°C. The Sanger sequencing method was performed to detect the PCR product's nucleotide with a specific forward and reverse primer. MYBPC3-A31P and A74T polymorphism was detected using Bioedit program and ApE (A plasmid Editor) program.

### Analysis of Peptide Patterns by MALDI-TOF MS

The protein concentration in serum was determined by the Lowry method (16). The absorbance at 750 nm (OD750) was measured, and the protein concentration was calculated using the standard curve, plotted between OD750 on Y-axis and BSA concentration (g/ml) on X-axis. The peptides from serum were acidified with 0.1% trifluoroacetic acid to the final concentration of 0.1 mg/ml. The peptides were mixed with MALDI matrix solution (10 mg sinapinic acid in 1 ml of 50% acetonitrile containing 0.1% trifluoroacetic acid), directly spotted onto MALDI target (MTP 384 ground steel, Bruker Daltonik, GmbH), and allowed to dry at room temperature. Maldi-TOF MS spectra were collected using Ultraflex III TOF/TOF (Bruker Daltonik, GmbH) in a positive linear mode with a mass range of 2,000–15,000 Da. Five hundred shots were accumulated with a 50 Hz laser for each sample. MS spectra were analyzed by using flexAnalysis and ClinproTool software (Bruker Daltonik, GmbH), including fingerprint spectra, pseudo-gel view, and principal component analysis (PCA). ACTH fragment 18–39 (human), Insulin oxidized B chain (bovine), Insulin (bovine), Cytochrome C (equine), and Apomyoglobin (equine) were used as external protein calibrations.

### Peptidomics Analysis by LC-MS

Peptide solutions were analyzed using an HCTultra PTM Discovery System (Bruker Daltonics Ltd., Germany.) coupled to an UltiMate 3000 LC System (Dionex Ltd., U.K.). Peptides were separated on a nanocolumn (PepSwift monolithic column 100 μm i.d. × 50 mm). Eluent A was 0.1% formic acid, and eluent B was 80% acetonitrile in water containing 0.1% formic acid. Peptide separation was achieved with a linear gradient from 10 to 70% B for 13 min at a flow rate of 300 nl/min, including a regeneration step at 90% B and an equilibration step at 10% B. One run took 20 min. Peptide fragment mass spectra were acquired in data-dependent AutoMS (2) mode with a scan range of 300–1,500 m/z, three averages, and up to 5 precursor ions selected from the MS scan 50–3,000 m/z.

For peptides quantitation, DeCyder MS Differential Analysis software (DeCyderMS, GE Healthcare) was used (17, 18). Acquired LC-MS raw data were converted, and the PepDetect module was used for automated peptide detection, charge state assignments, and quantitation based on the peptide ions signal intensities in MS mode. The analyzed MS/MS data from DeCyderMS were submitted for a database search using the Mascot software (Matrix Science, London, UK) (19). The data were searched against the NCBI database for protein identification. Database interrogation was; taxonomy (Canis lupus amilaris); enzyme (trypsin); variable modifications (carbamidomethyl, oxidation of methionine residues); mass values (monoisotopic); protein mass (unrestricted); peptide mass tolerance (1.2 Da); fragment mass tolerance (± 0.6 Da), peptide charge state (1+, 2+, and 3+) and max missed cleavages. The maximum value of each group was used to determine the presence or absence of each identified protein.

Data normalization and quantification of the changes in peptide abundance between the control and Maine Coon cats were performed and visualized using MultiExperiment Viewer (Mev) software version 4.6.1 (20). Briefly, peptide intensities from the LC-MS analyses were transformed and normalized using a mean central tendency procedure.

### Statistical Analysis

Data are presented as mean ± standard error of the mean (SEM). The normal distribution of data sets were conducted and then analyzed using one-way analysis of variance (ANOVA), a *P* < 0.05 was considered statistically significant.

## Results

Cats in the control group had the higher trend of age than the Maine Coon group (5.01 vs. 1.97 years, *P* = 0.065). In addition, there were significant differences by sex and weight between cats in the control and Maine Coon group. General characteristics and echocardiographic parameters for all cats are reported in Table 1. Blood profiles and serum biochemistry are reported in Table 2. Serum biochemistry (BUN/creatinine ratio) showed a statistically significant increase in control groups than cats in the Maine Coon group (*P* = 0.032). However, the levels of other biochemical profiles such as creatinine and blood urea nitrogen were not significantly different between groups. In addition, the blood profiles such as red blood cell count and hematocrit are not different in the control group vs. the Maine Coon group.

**Table 1:** Characteristics and echocardiographic variables of cats.

**Parameters** **(Mean ± SEM)**	**Control** **group**	**Maine Coon** **group**	**Reference** **value**
Age in years	5.01 ± 1.40	1.97 ± 0.40	*P* = 0.065
Weight (kg)	4.08 ± 0.30^**^	5.72 ± 0.27^**^	*P* < 0.001
Male (number [%])	6/17 [35 %]	8/14 [55 %]	–
LA/AO ratio	1.48 ± 0.05	1.50 ± 0.12	0.97–1.39
IVSd (cm)	0.58 ± 0.02	0.60 ± 0.03	0.347–0.621
LVPWd (cm)	0.56 ± 0.03	0.71 ± 0.05	0.343–0.634
LVIDd (cm)	1.21 ± 0.09	1.58 ± 0.08	1.076–1.883
IVSs (cm)	0.63 ± 0.02	0.73 ± 0.03	0.571–1.022
LVPWs (cm)	0.64 ± 0.03	0.78 ± 0.05	0.578–0.989
LVIDs (cm)	0.82 ± 0.07	1.02 ± 0.08	0.495–1.103
Fractional shortening (%)	42.50 ± 1.70	37.90 ± 3.00	39.9–64.3

*Values showed as mean ± SEM*,

** *P < 0.001*.

**Table 2:** Serum biochemistry, red blood cell count, and hematocrit in all cats and each groups (*P < 0.05).

**Parameters** **Mean ± SEM**	**Overall** **population**	**Control** **group**	**Maine** **Coon group**	**Reference** **value**
BUN (mg%)	36.44 ± 7.16	42.93 ± 11.67	24.28 ± 3.79	15–34
Creatinine (mg%)	2.01 ± 0.29	2.23 ± 0.47	1.61 ± 0.18	<2.0
BUN/Creatinine ratio	17.16 ± 0.99	18.48 ± 1.51*	14.68 ± 0.73*	7–37
HGB (gm%)	11.22 ± 0.60	11.21 ± 0.81	11.25 ± 0.91	10–15
PCV (%)	33.94 ± 1.71	34.79 ± 2.38	32.15 ± 2.44	30–45
RBC (×10^6^/ul)	7.42 ± 0.43	7.27 ± 0.64	7.74 ± 0.54	5–10
WBC	13.88 ± 1.06	13.27 ± 1.66	15.18 ± 0.90	5.5–19.0

**Figure 2:**
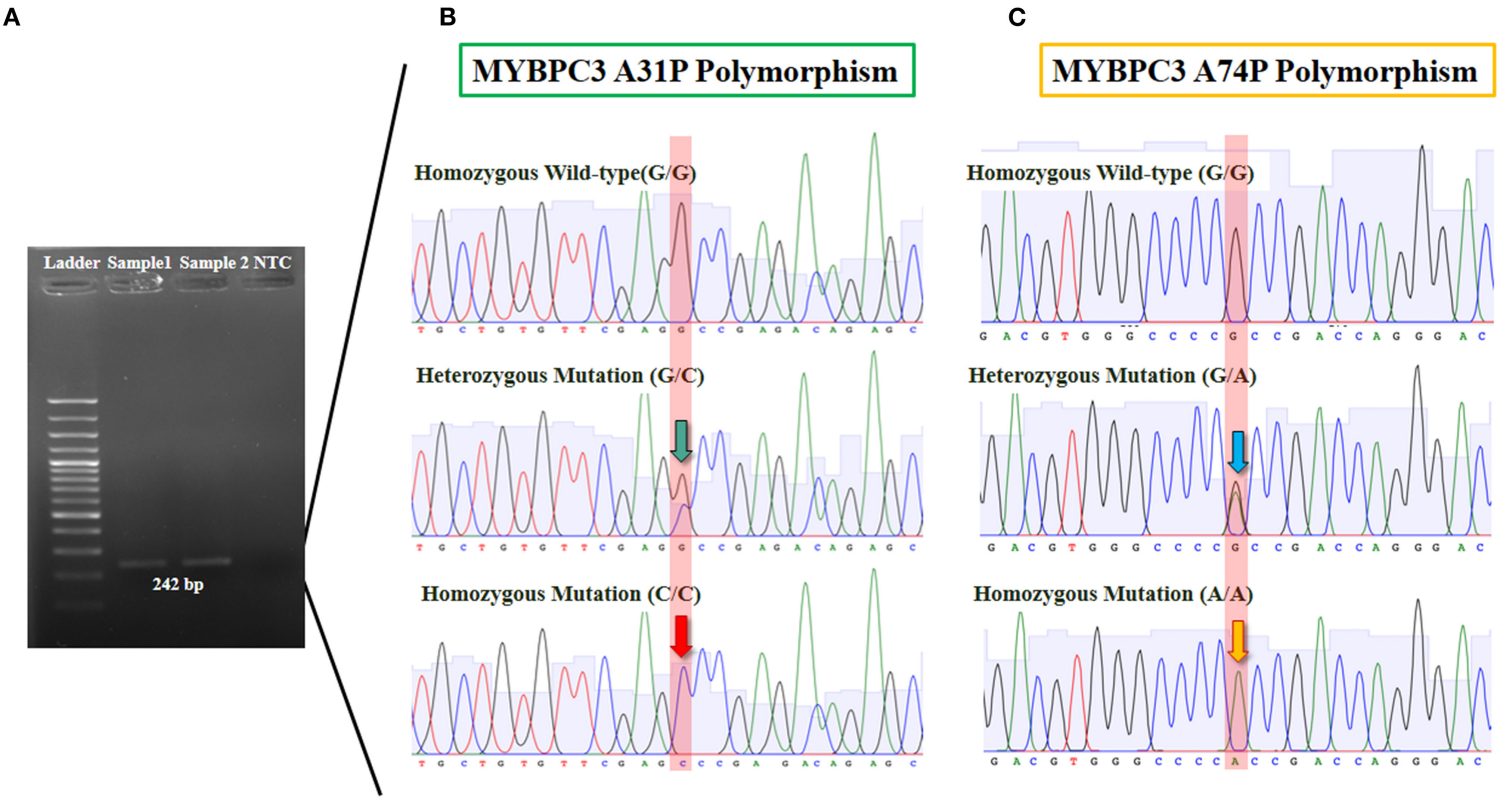
The results of MYBPC3-A31P and A74T polymorphism from genotyping by sequencing. **(A)** Gel electrophoresis was represented 242 bp of target DNA of MYBPC3 gene in lane 2 and 3. **(B)** The sanger sequencing result of MYBPC3-A31P was showed in three group including homozygous wild-type (G/G), heterozygous mutation (G/C), and homozygous mutation (C/C). The green arrow pointed 2 peaks of guanine and cytosine and the red arrow pointed of only cytosine. **(C)** MYBPC3-A74T sequencing result was showed in three group including homozygous wild-type (G/G), heterozygous mutation (G/A), and homozygous mutation (A/A). The blue arrow pointed 2 peaks of guanine and alanine while the yellow arrow represented one peak of alanine.

Analysis of the association between Maine Coon cats and genotype at A31P and A74T mutation was performed. Results showed significant differences between 78.57% (*n* = 11) and 21.43% (*n* = 3) genotypes for the homozygous wildtype and heterozygous mutation, respectively (Figure 2).

**Figure 3:**
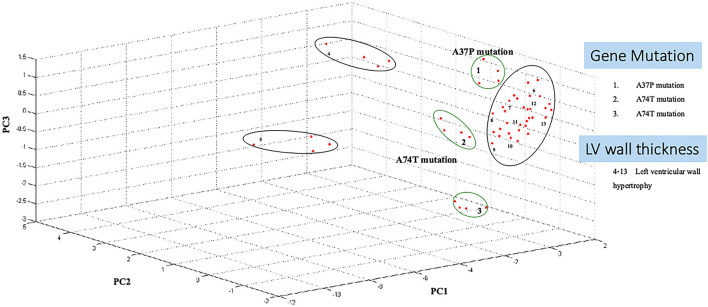
Principal component analysis shows Maine Coon cats samples segregate from control cats. A three-dimensional scatterplot plot of non-gene mutation, gene mutation (A31P and A74T), and left ventricular wall hypertrophy cats (LV wall Thickening).

**Figure 4:**
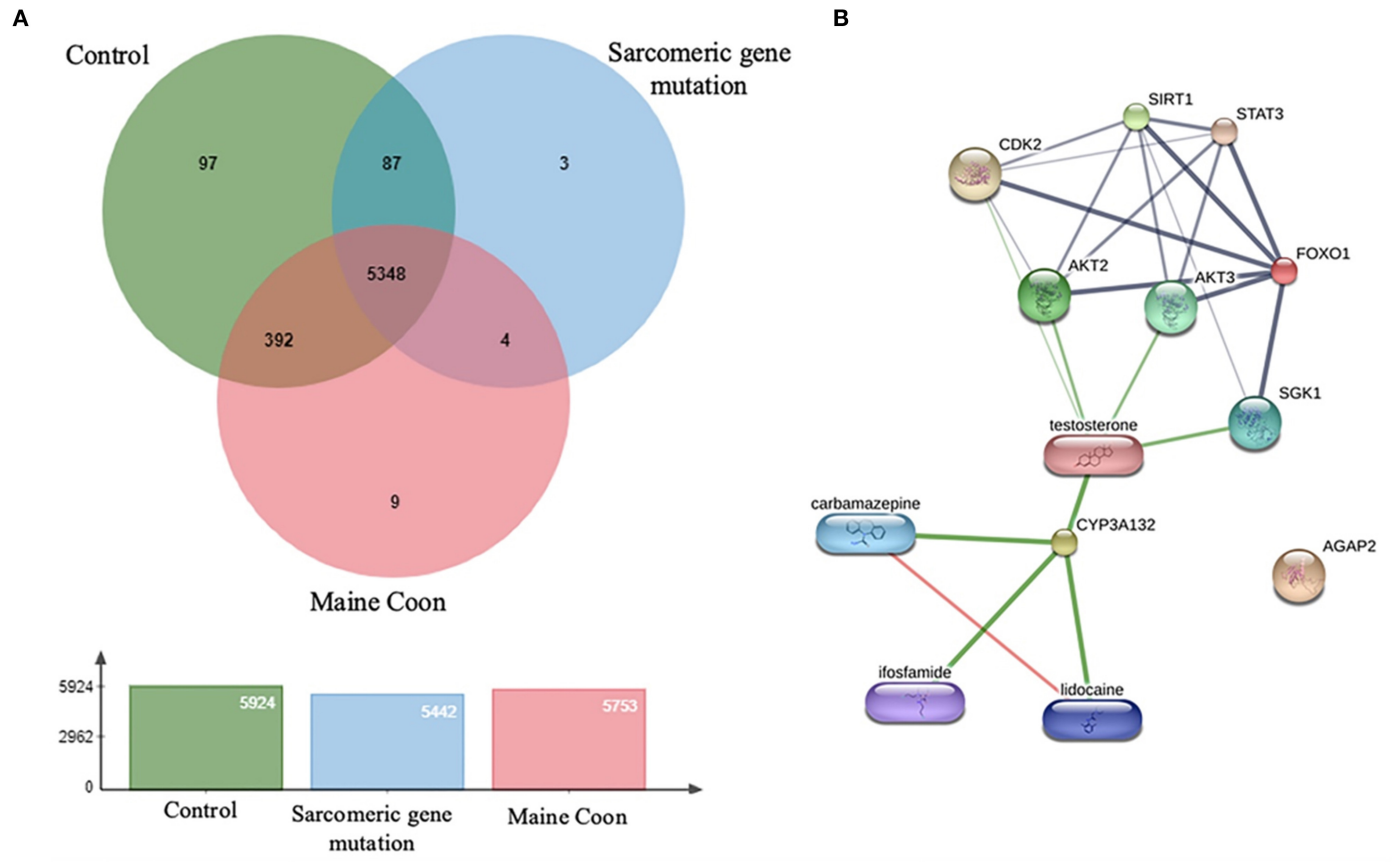
**(A)** The diagram represents overlap among protein types with 5,946 identified proteins. **(B)** Protein and drug interaction networks of proteins dominate in sarcomeric gene mutation cats.

Three-dimension component analysis showed distinct clusters among the sarcomeric gene mutation, non-mutation cats, and normal control groups. All 31 replicates from each pooled serum sample group exhibited a distinguished cluster from the others, indicating a distinctive peptide profile in each group and demonstrating the uniformity and homogeneity of data within the groups as showed in Figure 3. In the present study, peptidomics analysis showed a total of 5,946 peptides differentially expressed between groups (Figure 4). STITCH version 5.0 was used to determine the protein names and interactions. Results showed that eight peptides were found to be up-regulated in the Maine Coon group including, IQ motif and Sec7 domain 2 (IQSEC2), zinc finger NFX1-type containing 1 (ZNFX1), discs large (Drosophila) homolog-associated protein 1 (DLGAP1), CTS telomere maintenance complex component 1 (CTC1), opiate receptor-like 1 (OPRL1), aryl hydrocarbon receptor nuclear translocator-like (ARNTL), nudix (nucleoside diphosphate linked moiety X)-type motif 16 (NUDT16), and KH domain containing RNA binding signal transduction associated 2 (KHDRBS2). In addition, three peptides were up-regulated in the Maine Coon group with sarcomeric gene mutation containing, cytochrome 3a132 (CYP3A132), forkhead box O1 (FOXO1), and ArfGAP with GTPase domain, ankyrin repeat and PH domain 2 (AGAP2).

The proteins were identified using Gene Ontology Annotation Database. The expression of hepatic markers such as CYP3a132 was investigated in the present study, CYP3a132 has been reported to associate with liver disease in domestic cats (21). In addition, gene mutation cats were associated with specific antioxidant defense changes in a distinct functional group of proteins. The different expression peptides were from a wide range of functional classes and included proteins involved in signal transduction, such as FOXO1. Transcription factor FOXO1 plays an essential role in glucose metabolism, cell cycle progression, apoptosis, and differentiation (22).

**Figure 5:**
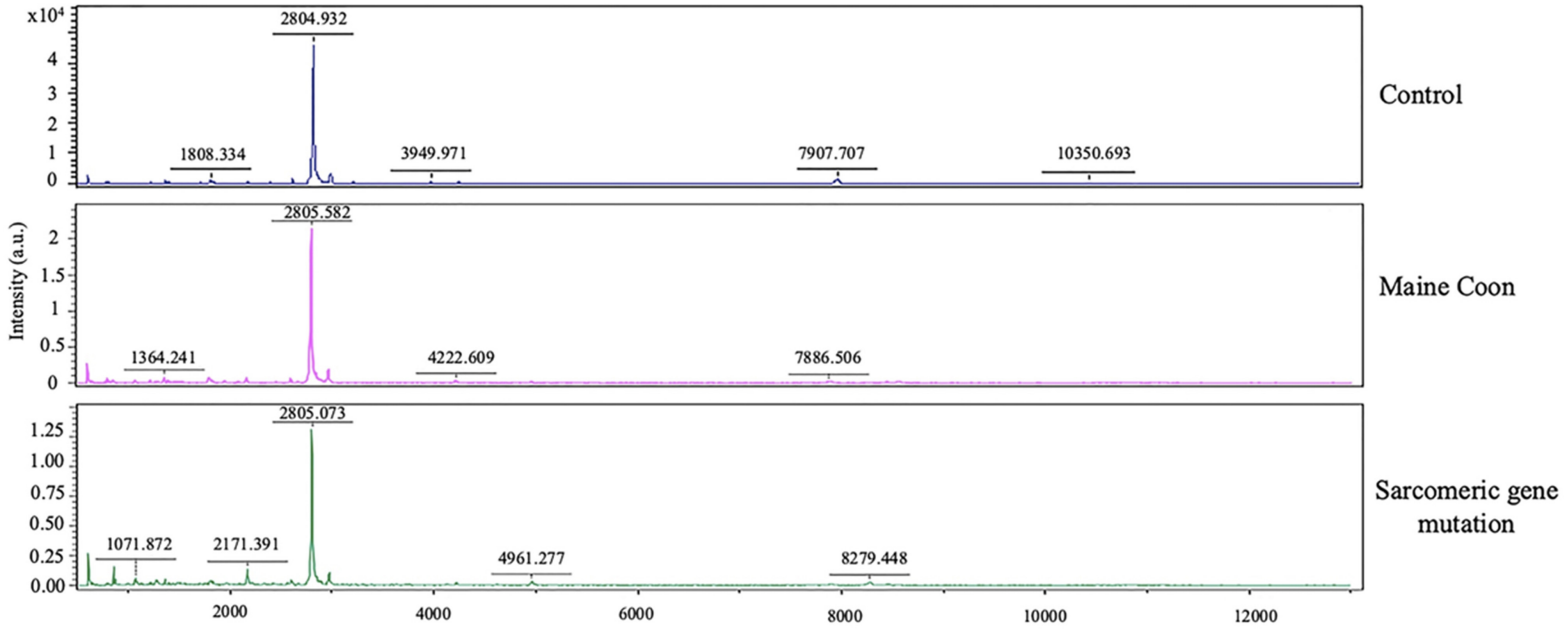
Protein identification by MALDI-TOF analysis. Mass spectrometry of the protein isolated from the 2D gels of protein extracts of cat serum in domestic shorthair cats (Control), Maine Coon cat, and Maine Coon with sarcomeric gene mutation cats.

Moreover, AGAP2 was another peptide that expressed in the gene mutation Maine Coon cats. AGAP2 seems to be involved in TGFβ1 signaling that could contribute to the progression of hepatic fibrosis, suggesting AGAP2 as a potential new molecular target for liver fibrogenesis (23). Moreover, the peptides of gene mutation in cats were associated with testosterone, analgesic, anticonvulsant, and anticancer drugs, as shown in Figure 4. Verification of expressed protein sequences by LC-MS, the peptides mass peaks were compared among groups (Figure 5).

## Discussion

In this study, the differential peptidomic profiles expressed in Maine Coon cats compared to control cats may provide complementary tools for detecting and evaluating hypertrophic cardiomyopathy. Results showed the protein and drug interaction networks of proteins that dominate in sarcomeric gene mutation cats (Figure 4B). Since diseases are often a consequence of multiple changes in the same pathway or protein complex, the role of proteins in biological systems can be understood with the function of the targeted small molecules or drugs. Chemicals or drugs in the interaction network predicted by STITCH can lead to a better understanding of the potential function of the interacting protein partners. Among the expressed peptides, three peptides were increased in Maine Coon cats with sarcomeric gene mutations compared to nonmutated cats. The findings in our study are consistent with previously reported findings that the proteins involved in glucogenesis and oxidative phosphorylation are closely related to cardiac hypertrophy. The proteins identified in the cat with sarcomeric gene mutations included FOXO1 and CYP3A132. These proteins have an important role in several conditions associated with testosterone and glucose metabolic control. Testosterone has been reported to stimulate glucose metabolism by activating AMP-activated protein kinase (AMPK) and androgen receptor (AR) signaling, which are critical for induction of cardiomyocyte hypertrophy. Our results suggest that inhibiting AMPK and AR may help block glycolysis and cardiomyocyte hypertrophy, similar to the findings in previous reports (14). However, further studies should be performed to confirm this result.

FOXO1 is a transcription factor that modulates cell apoptosis and cell differentiation (24). It has been reported that TGFβ1 regulates the overexpression of FOXO1 as a consequence of inducing cardiac myoblast differentiation to myofibroblasts (25). Thus, FOXO1 may be related to the pathway of cardiac fibrosis and cardiac hypertrophy in Maine Coon cats with sarcomeric protein mutations. Similar to the findings in chronic intermittent hypoxia, FOXO1 induces upregulated expression of an apoptosis-related gene (Bim) and caspase 9, leading to cardiac hypertrophy in obstructive sleep apnea syndrome (26). Furthermore, this finding agrees with previous research showing that the regulation of FOXO1 is correlated with apoptosis-related genes such as Bcl-2 and Bim (27). A report in 2020 revealed that FOXO1 knockdown or deletion reduces cardiac hypertrophy caused by pressure overload (28).

AGAP2 is a GTPase-activating protein associated with numerous signaling pathways, including cell survival, cellular migration, and cell apoptosis. Evidence has shown that AGAP2 regulates profibrotic properties in liver fibrosis *via* TGFβ1. In addition, elevated AGAP2 acts as a pathological factor of cancer and liver fibrosis (29). According to the results in this study, AGAP2 might be correlated with cardiac fibrosis and hypertrophy in Maine Coon cats with HCM. However, reports linking AGAP2 with cardiac hypertrophy in cats still require further confirmation.

CYP3A132 belongs to the cytochrome P450 3A (CYP3A) family and is normally expressed in the liver (21). A previous study reported that CYP3A132 could be used as a marker for liver fibrosis. CYP3A132 has been found in domestic cats and is related to biotransformation and therapeutic drug metabolism, including that of calcium channel blockers, benzodiazepines, and immunosuppressant drugs (21). Moreover, subclinical liver fibrosis is associated with a history of atrial fibrillation, heart failure, and congestive heart disease (30). CYP3A132 was expressed in Maine Coon cats with sarcomeric gene mutations compared with nonmutated cats. Therefore, CYP3A132 may be used as a marker to detect cardiac hypertrophy in cats. However, further studies are warranted to determine the relationship between these peptides and HCM in cats.

According to the crucial serum biochemistry, thyroxine hormone concentration and feline NT-proBNP plasma concentration should use as a screening tool for cats with subclinical hypertrophic cardiomyopathy (HCM). These factors can influence the inclusion criteria in our study. Unfortunately, in this study, these factors were not measured, which is our study's limitation. However, our study used transthoracic echocardiography to measure left ventricular wall thickness is considered the reliable criteria in classifying HCM as recommended in the ACVIM guidelines (31).

## Conclusion

The MYBPC3-A31P and A74T mutations had a high frequency in Maine Coon cats. A mutation was associated with the hypertrophic cardiomyopathy phenotype. Peptidomic testing of Maine Coon cats will be a helpful tool for identifying carriers of the mutated gene for myocardial disease in cats. The results from this study may provide important information on clinical presentation, protein expression, and a gene associated with feline HCM.

## Data Availability Statement

The original contributions presented in the study are included in the article/supplementary materials, further inquiries can be directed to the corresponding author/s.

## Ethics Statement

The animal study was reviewed and approved by the Animal Care Committee of Kasetsart University, Approval Number ACKU-62-VET-059. Written informed consent was obtained from the owners for the participation of their animals in this study.

## Author Contributions

SP and PS wrote the original manuscript and prepared figures. SR, ST, NP, and SP analyzed and interpreted the data regarding the serum peptidomics profiles. PS performed the PCR and DNA sequencing. SP was a major contributor in writing the manuscript. All authors read and approved the final version of the manuscript.

## Funding

This research project was supported by National Research Council of Thailand (NRCT): NRCT5-RGJ63002-035.

## Conflict of Interest

The authors declare that the research was conducted in the absence of any commercial or financial relationships that could be construed as a potential conflict of interest.

## Publisher's Note

All claims expressed in this article are solely those of the authors and do not necessarily represent those of their affiliated organizations, or those of the publisher, the editors and the reviewers. Any product that may be evaluated in this article, or claim that may be made by its manufacturer, is not guaranteed or endorsed by the publisher.
